# Liquid Phase Micro-Extraction of Linear Alkylbenzene Sulfonate Anionic Surfactants in Aqueous Samples

**DOI:** 10.3390/membranes1040299

**Published:** 2011-10-13

**Authors:** Niklas Larsson, Paulina Otrembska, Mercedes Villar, Jan Åke Jönsson

**Affiliations:** 1 Division of Analytical Chemistry at Lund University, P.O. Box 124, Lund 221 00, Sweden; E-Mail: jan_ake.jonsson@organic.lu.se; 2 University of Opole, Oleska 48, Opole 45–050, Poland; E-Mail: paulina.mizera@gmail.com; 3 Department of Analytical Chemistry, Faculty of Chemistry, University of Seville, C/Professor García González s/n, Seville 41012, Spain; E-Mail: mvn@us.es

**Keywords:** linear alkylbenzene sulfonate, extraction, liquid-phase microextraction, ion-pairing, method development, environmental analysis, surfactant

## Abstract

Hollow fiber liquid phase micro-extraction (LPME) of linear alkylbenzene sulfonates (LAS) from aqueous samples was studied. Ion pair extraction of C_10_, C_11_, C_12_ and C_13_ homologues was facilitated with trihexylamine as ion-pairing agent, using di-*n*-hexylether as solvent for the supported liquid membrane (SLM). Effects of extraction time, acceptor buffer concentration, stirring speed, sample volume, NaCl and humic acids were studied. At 10–50 μg L^−1^ linear R^2^-coefficients were 0.99 for C_10_ and C_11_ and 0.96 for C_12_. RSD was typically ∼15%. Three observations were especially made. Firstly, LPME for these analytes was unusually slow with maximum enrichment observed after 15–24 h (depending on sample volume). Secondly, the enrichment depended on LAS sample concentration with 35–150 times enrichment below ∼150 μg L^−1^ and 1850–4400 times enrichment at 1 mg L^−1^. Thirdly, lower homologues were enriched more than higher homologues at low sample concentrations, with reversed conditions at higher concentrations. These observations may be due to the fact that LAS and the amine counter ion themselves influence the mass transfer at the water-SLM interface. The observations on LPME of LAS may aid in LPME application to other compounds with surfactant properties or in surfactant enhanced membrane extraction of other compounds.

## 1. Introduction

Linear alkylbenzene sulfonates (LAS) are primarily used as detergents in household applications [1,2]. In 2005, 430 kilotons of LAS were consumed in Europe [1], making LAS one of the major classes of anionic surfactants on the market, [1,3] representing more than 41% of the consumed amount [4]. Commercial LAS is a mixture of homologues and isomers with a sulfonated benzene ring *para*-substituted to a linear alkyl chain (usually between 10 and 13 carbon atoms) except at the terminal carbons [1]. With increasing homologue number, surface activity increases [2]. Both toxicity and bioconcentration factor [5] as well as ease of biodegradation [6] are dependent on homologue number and isomer. LAS are well degraded under aerobic conditions and their concentration in sewage treatment plant (STP) effluent is usually more than 98% lower than inlet concentrations [1,7,8], while degradation is less efficient under anaerobic conditions [1,8,9]. Typical LAS concentrations in STP effluent are >0.07 mg L^−1^ and in recipients 0.01 mg L^−1^ or less [2]. Risk assessment quotients (predicted environmental concentration divided by the predicted no-effect concentration) of 0.17 for aqueous compartments and 0.65 for sediment have been reported [1], so environmental risks of LAS are not acute. However, there are surfactants that are less efficiently removed in STPs than LAS, e.g., alkylphenol ethoxylates or cationic surfactants [7]. Further, new surfactants (e.g., fluorinated ones), put new demands on analytical techniques [10,11]. Overall, these reasons motivate development of sample preparation methods for surfactants.

For anionic surfactants in aqueous matrices, the methylene blue active substances (MBAS) method is a standardized technique [12,13], but this method is non-selective, sensitive to interferences and it cannot distinguish between homologues [13]. SPE has often been used in sample preparation of LAS [7,8,14,15,16,17,18,19]. With solid phase microextraction (SPME), enrichment factors ∼10 times and limits of detection (LOD) of individual homologues ∼0.5 μg L^−1^ have been reported [20]. Recently, SPME enrichment factors about 1000 times and LOQs ∼1 μg L^−1^ were achieved, but equilibration times up to 33 h were needed [21].

An extraction technique which may decrease the labor needed in SPE and simultaneously increase enrichment factors compared with SPME is membrane extraction. Here, a supported liquid membrane (SLM) is formed by impregnating a porous membrane with an organic liquid, which is immobilized by capillary forces in the membrane pores. With an aqueous phase on each side (sample and acceptor side, respectively) of the SLM, the system is in principle a miniaturized liquid-liquid extraction (LLE) system involving both forward and backward extraction [22]. With hollow fibers (HF) as supporting material, such extraction is often referred to as liquid-phase microextraction (LPME) [23]. In this work, the application of LPME for extraction of LAS was studied.

Since LAS is negatively charged at all pH > 0, it is necessary to perform membrane extraction of LAS as ion-pair extraction [24]. This can be done by ion-pairing LAS with a positively charged counter ion, thus forming a neutral extractable ion-pair [22,23,24,25]. If an amine is employed as ion-pairing agent the sample pH shall be set so the amine is positively charged and the acceptor pH should be set so that the amine becomes neutral. The latter step breaks the ion pair and traps the analyte in the acceptor [24]. As the extraction proceeds, this trapping mechanism leads to enrichment of the analyte.

SLM extraction of LAS (in a flow system) was studied by Miliotis *et al.* [24]. Their work set the basis for the current work. Of three tested organic liquids, di-*n*-hexylether (DHE) performed best as organic solvent for the SLM [24]. Of four ion-pairing agents (amines), trihexylamine (THA; p*K_a_* ∼10.46 [26]) was optimal and was used at 100 mg L^−1^ in samples buffered at pH 7. In the current work based on LPME, the same SLM solvent and counter ion was used, assuming that THA and DHE would perform well also in LPME. Due to adsorption and carry-over problems, sodium dodecylsulfate (SDS) was added to the sample and acceptor buffers at 200 mg L^−1^, giving overall more efficient extraction [24]. Typically, extractions lasted 40–50 min and enrichment factors about 40 times were achieved. A slow mass transfer of LAS over the membrane/acceptor interface was noted and the mass transfer of LAS was determined to be membrane-controlled [24], *i.e.*, the rate-limiting step is in the membrane and the extraction is governed by the partition ratio into and/or diffusion through the membrane [27].

## 2. Experimental Section

### 2.1. Chemicals and Solutions

A dodecylbenzenesulfonic acid sodium salt (CAS 25155–3-0) product mix of homologues and isomers, with ∼80% LAS (cat no. D-2525) obtained from Sigma-Aldrich (Steinheim, Germany), was used. Trihexylamine (THA) and di-*n*-hyxylether (DHE) (>97%, purum) were obtained from Fluka Chemical AG (Buchs, Switzerland). THA >97% was used for initial studies while purum grade (>99%) was used for quantitative experiments. Acetonitrile, Chromasolv®), SDS (>99%) and humic acids (no. H1,675–2) were obtained from Sigma-Aldrich. Formaldehyde solution (>37%), HCl (37%), Na_2_HPO_4,_ NaH_2_PO_4_ and NaClO_4_ were obtained from Merck (Darmstadt, Germany). NaOH (reagent grade) was obtained from Scharlau Chemie S.A (Barcelona, Spain). NaCl of analytical/pro analysi grade from both Fisher Scientific (Loughborough, UK) and Merck were used. Stock solutions were prepared at 1 g L^−1^ LAS in methanol. THA stock solutions were prepared at 100 mg L^−1^ in 0.1 M phosphate buffer at pH 7. SDS was dissolved in 50% water and 50% methanol. All water used in preparation of solutions was purified with a Milli-Q-RO4 system (Millipore, Bedford, MA, USA).

The glassware was burned 10–20 h at 200 °C, cleaned with methanol and kept aside for this project. Between each extraction, sample flasks were cleaned with water and methanol and weekly burned in order to minimize carry-over. Acceptor solutions were NaOH at pH 12 or phosphate buffer adjusted with NaOH to pH 12. Acceptor and calibration solutions were prepared with 200 mg L^−1^ SDS in order to decrease analyte adsorption. Sample buffers at pH 7.0 were prepared by mixing 10 mM Na_2_HPO_4_ with 10 mM NaH_2_PO_4_. A model 211 microprocessor pH meter (Hanna Instruments) was used in the preparation of sample, acceptor and mobile phase buffers. For measuring acceptor pH (in some cases), a Ross 8220BNWP (Thermo Fisher Scientific, Beverly, MA, USA) microelectrode was used.

### 2.2. LPME

Q3/2 Accurel PP polypropylene hollow fiber membranes (200 μm wall thickness, 600 μm ID, 66% porosity, 0.2 μm pore size) were obtained from Membrana GmbH (Wuppertal, Germany). Fibers were cut with a scalpel into 3.7 cm long pieces. One end was closed by heating. Fibers were washed with acetone under sonication, dried and kept in a closed Petri dish until use. After this preparation, an HF had an effective length of ∼3.5 cm and could fit ∼10 μL acceptor in the lumen. With a microliter syringe, acceptor buffer was filled into the HF until aqueous solution was seen exiting the pores. The fiber was dipped into DHE for a few minutes to impregnate the pores of the HF wall and form the SLM. To wash surplus solvent from the HF surface, the HF was immersed into water and either shaken up to 30 s or sonicated ∼1 s.

The HF was held in the sample using two slightly different setups. In initial studies, the syringe used to fill the acceptor was carefully replaced with a solid metal rod (soldering tin or stainless steel). The rod could easily be bent so that the HF could be adjusted by the edge of an Erlenmeyer flask or in the neck of a volumetric flask, assuring the HF was below the solution surface and did not interfere with the stir bar magnet. However, this setup had disadvantages, such as increased labor and risk for loss of acceptor, which could occur when the syringe was taken off, the metal rod was inserted or removed or when the syringe was connected to collect the extract (described below). The setup with metal rods was used in studying the effects of counter-ion concentration, extraction time, acceptor composition, sample volume, stirring speed, sodium chloride and application to tap and surface waters, but was eventually replaced. With more microliter syringes at hand, the HF needed not to be removed from the syringe that was used to fill the acceptor. With this setup, i.e. the usual setup for LPME [23,28], the sample was kept in a volumetric flask and the HF was placed a few mm under the solution surface in the neck of the flask. The syringe with the HF was held in place by a laboratory clamp. The syringe setup was used in studying the effects of stirring speed, extraction linearity and matrix effects, such as effects of humic acids and application to surface water.

During extraction, samples were stirred with an IKAMAG RO10 power stirrer (IKA-Werke, Staufen, Germany) with place for 10 samples for simultaneous treatment and maximum speed of 1100 rpm. After extraction, the HF was removed from the sample and quickly blotted with a Kleenex tissue to remove drops on the HF surface. The sealed end of the fiber was cut. The acceptor was withdrawn with a syringe (in applicable cases with the syringe that held the HF during the extraction) and the volume (typically 9–11 μL) was noted. The extract was placed in a vial with a conical insert. For column compatibility, the extract was neutralized with 0.1 M HCl. The volume of HCl was 50% of the extract volume. Capped vials were stored in refrigerator or analyzed directly. To avoid cross contamination, the syringe was washed 3 times in acceptor buffer followed by 3 times in methanol between extracts.

Experiments were evaluated by calculation of extraction efficiencies (*E*) and enrichment factors (*E*_e_). *E* is defined as *n*_A_/*n*_S_, where *n* is the total analyte amount in the sample (subscript S) or in the acceptor (subscript A). *E*_e,_ is defined as *C*_A_/*C*_S_. *C*_S_ is the nominal spiked sample concentration and *C*_A_ the acceptor concentration. Relative matrix effects are described by comparing the enrichment factor in a sample with the studied matrix component (e.g., humic acids) to that of a sample without this matrix component by division, *i.e.*, *E*_e-matrix relative_ = *E*_e-with matrix_/*E*_e-without matrix_.

### 2.3. HPLC System and Quantification

The employed HPLC system was an Agilent 1100 liquid chromatograph (Agilent, Santa Clara, CA, USA). 5 μL was injected with the autosampler. Carry-over was eliminated by washing the needle after each injection with acceptor buffer containing 200 mg L^−1^ SDS. A C_18_ column (3 μm, 4.6 mm ID × 150 mm (ACE ®, Aberdeen, Scotland)) was used, giving the possibility to separate both homologues and isomers [19,29,30], even though keeping the isomers unresolved with C_8_ columns can increase the homologue signal relative to the baseline [31]. Different mobile phases for LAS separation have been presented, whereof various mixtures of an aqueous phase and acetonitrile have frequently been used [16,17,19,29,30], sometimes with sodium perchlorate [16,19,29] and sometimes in gradient mode [16,17,19]. Separation was performed with acetonitrile and phosphate buffer (5 mM, pH 6). 0.1 M sodium perchlorate was used as modifier to increase resolution [29]. The flow was 0.5 mL min^−1^ and the column was thermostated to 25 °C. The separation program developed here was as follows: constant 50% for 1 min, gradient to 60% acetonitrile until 10 min, and constant until 28 min. The column was then washed with a 1 min gradient to 70% acetonitrile and kept constant at 70% during at least 2 min, followed by restoration to initial conditions during 1 min and constant at 50% for 1 min. The Agilent 1100 FLD module was used for fluorescence detection. Following the FLD optimization procedure described by Agilent, 230 nm was used for excitation and 310 nm for emission.

Calibration solutions were prepared by dilution of the LAS product with acceptor buffer. The LOD of the HPLC was ∼200 μg L^−1^. The HPLC was calibrated in two intervals. The low interval was nominally 0.2–30 mg L^−1^ with the FLD photo-multiplier set to the maximum of 18 (arbitrary units). The high interval was 80–900 mg L^−1^ with the photo-multiplier set to 13. Isomer peaks were integrated individually, but were summed to obtain the total signal of each homologue, giving linear R^2^-coefficients of 0.995–0.999. The fractions of homologues in the employed LAS product were estimated to 13% C_10_, 24% C_11_, 24% C_12_ and 19% C_13_, using the 0.2–30 mg L^−1^ calibration and assuming equal FLD sensitivity for all isomers as well as exactly 80% of total LAS mass concentration. These fractions were taken into account to estimate LOD for the individual homologues. For calculation of *E* and *E*_e_, these fractions were not taken into account, since sample, acceptor and calibration solutions were all expected to be equally proportionally lower. Unless otherwise noted, presented LAS concentrations refer to nominal concentrations.

### 2.4. Sampling of Surface Water

Effluent samples were collected in the wintertime from Källby STP (Lund, Sweden) at the beginning of the third denitrification pond. Recipient river [32] samples were collected ∼1.4 km upstream and ∼1.5 km downstream the plant, respectively. Clean bottles (1–2 L) were dipped upside down into the water and turned toward the direction of water flow. Samples were conserved by adding formaldehyde to a final 1% (*v*/*v*) concentration and stored at 4 °C in darkness less than 4 days [12]. In total 54 mL formaldehyde was added per 2 L sample.

## 3. Results and Discussion

### 3.1. Concentration of Ion-Pairing Agent

In order to maximize enrichment, the ion-pairing amine should be in excess. For extraction of 1 mg L^−1^ LAS, the tested THA concentrations were 10, 50, 75 and 100 mg L^−1^ (*n* = 3). 100 mL samples were extracted during 24 h with 330 rpm stirring and 0.01 M NaOH was used as acceptor. For 50 and 75 mg L^−1^, precision was poor. For 100 mg L^−1^, *E*_e_ was significantly lower than for 10 mg L^−1^. Possibly, the highest concentration of THA had a solvating of effect on the SLM during long extractions. 10 mg L^−1^ THA was considered optimal.

### 3.2. Extraction Time and Acceptor Composition

**Figure 1:**
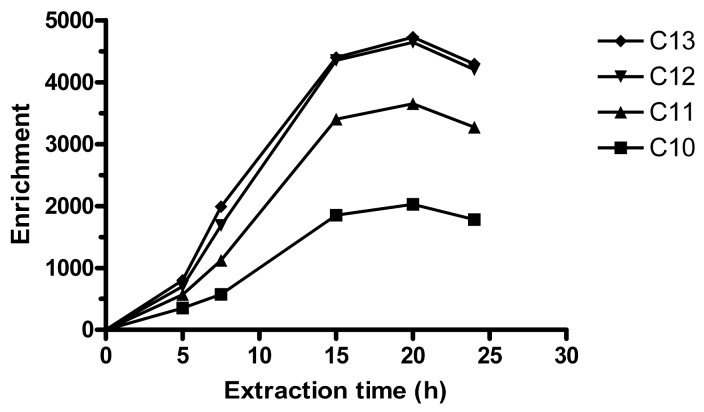
Enrichment as a function of extraction time for 65 mM phosphate buffer at pH 12 (*n* = 3). Nominal linear alkylbenzene sulfonates (LAS) sample concentration was 1 mg L^−1^, trihexylamine (THA) concentration 10 mg L^−1^, 250 mL sample in volumetric flask stirred at 770 rpm.

An equilibrium time of 15 h can be observed in Figure 1. In the equilibrium regime, the enrichment is determined by a distribution ratio between the acceptor and the sample, and extraction time is not critical. Unless otherwise noted, 15 h was employed for further experiments. For some sets of experiments, 20 h was used. These extraction times are longer than in typical SPE methods, but shorter than for SPME [21]. The LPME extraction time is relatively long, but with the magnetic stirring table used, up to 10 replicates could be extracted simultaneously overnight and analyzed by HPLC the following day.

Parallel to the extraction of LAS:THA ion pairs, there is also a continuous transport of H^+^ to the acceptor, since the amine is transported in charged form. Eventually, this decreases the acceptor pH and reduces the driving force for the extraction. Previously, 0.01 M NaOH was used as acceptor in SLM extractions, which lasted ∼40–50 min [24]. However, using HFs and employing longer extraction times, *E*_e_ dropped after 1–5 h. Therefore, 32.5 mM phosphate buffer adjusted to pH 12.0 was used, which gave more stable pH and *E*_e_. By doubling the buffer concentration to 65 mM, higher *E*_e_ were obtained, which gave the extraction equilibrium in Figure 1. 130 mM phosphate buffer was also tested (*n* = 3 at 15 h), but this lead to that *E*_e_ dropped with ∼85% for all homologues. Possibly, a buffer with lower ionic strength and density could have improved extraction more than phosphate, due to lower salting out-effect and faster diffusion. Here, 65 mM phosphate buffer was considered optimal.

As is noted in Figure 1 the extraction is slow. Typically, equilibrium times in LPME are faster and range from ∼30 min [23,33] to 6–7 h [25]. A slow transfer of LAS over the membrane/acceptor interface was previously suspected in SLM extraction of LAS [24]. The long extraction time observed here is also similar to the slow extraction in ion-pair LPME of a cationic amine surfactant [34]. The extraction of cationic surfactant was performed in a 2-phase system, which lacked the SLM/acceptor interface. This suggests that the rate-limiting step in ion pair HF-LPME of surfactant is perhaps either in the transfer of analyte across the sample/SLM phase boundary or into the bulk of the SLM, and possibly not at the interface between the SLM and the acceptor.

Slow extraction could possibly be due to extraction of reversed micelles. The positive counter ion could balance the repulsive effect between sulfonate head groups. Reversed micelle extraction of proteins requires extraction times of 24 h [35] to 100 h [36]. In surfactant enhanced LPME of drugs, maximum enrichment was reached after ∼40 min, but it was found that when the sample concentration of (nonionic) surfactant exceeded the critical micelle concentration, *E* decreased sharply [33]. The interpretation was that drugs were incorporated into the micelles, which could not completely pass the HF pores. However, data on reversed micelles is more scarce than of micelles in aqueous solution [3]. Micelle formation in water usually occurs over a limited concentration range, while physical properties of non-aqueous solutions related to (reversed) micelle formation often undergo a continuous transition over orders of magnitude in concentration [3].

### 3.3. Stirring Speed and Sample Volume

**Figure 2:**
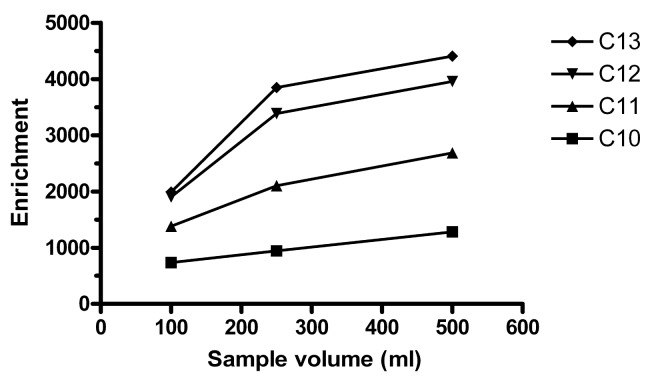
Effect of sample volume. Conditions as in Figure 1, except 32.5 mM acceptor buffer concentration. *n* = 2 for 100 and 500. *n* = 3 for 250 mL.

More vigorous stirring of the sample increases the contact between the sample and the HF, decreases the boundary layer on the HF outside and thus increases enrichment. 330, 770 and 1100 rpm were tested in 100, 300 and 500 mL samples extracted in E-flasks (*n* = 1 for each speed:volume combination). For 1100 rpm, *E*_e_ was very low or the extract was washed out, which was noticed when it should be collected with a syringe. The highest *E*_e_ was observed for 300 mL stirred at 770 rpm, followed by 500 mL at 770 rpm. In volumetric flasks, 250 and 500 mL gave about the same *E*_e_ (Figure 2), but the precision was much better for 250 mL (RSD of 2–13% for the different homologues) than for 500 mL (RSD 30–67%). In retrospect, too few replicates may have been performed for 500 mL. If one of the extracts for 500 mL was a statistical outlier, this would have decreased precision. This experiment was performed with the setup using metal rods and not syringes holding the HFs during the extraction. 250 mL was considered optimal at that time and thereafter used throughout.

It was observed that precision depended on how much the HF moved as the sample was stirred. In 100 mL volumetric flasks, 550 rpm was the lowest stirring speed that did not cause the HF to shake in an unstable way. Table 1 compares extraction between 250 mL × 770 rpm and 100 mL × 550 rpm. Typically, increasing the sample volume in membrane extraction increases the enrichment, *E*_e_, but decreases the fraction of extracted analyte from the sample, *E*. This could also be observed here. However, for the lower homologues C_10_ and C_11_, *E* was higher for the larger sample. It can also be noted that the difference in both *E*_e_ and *E* was lower for all homologues for 250 mL than for 100 mL. When the fiber is exposed to less analyte, higher homologues are favored. Whether this is at the expense of the lower homologues is unclear.

**Table 1:** Effects of sample volume and stirring speed (relative standard deviations (RSD) in brackets). Time was 15 h for 250 mL and 20 h for 100 mL. Other conditions as in Figure 1.

**Homologue**	**Enrichment factor (*E*_e_)**		**Extraction efficiency (*E*, %)**	

	**250 mL × 770 rpm (*n* = 3)**	**100 mL × 550 rpm (*n* = 3)**	**250 mL × 770 rpm**	**100 mL × 550 rpm**
C_10_	1852 (20%)	560 (11%)	11	6
C_11_	3403 (18%)	1523 (12%)	20	15
C_12_	4352 (16%)	2913 (13%)	25	29
C_13_	4400 (14%)	3597 (16%)	26	36

### 3.4. Linearity

**Figure 3:**
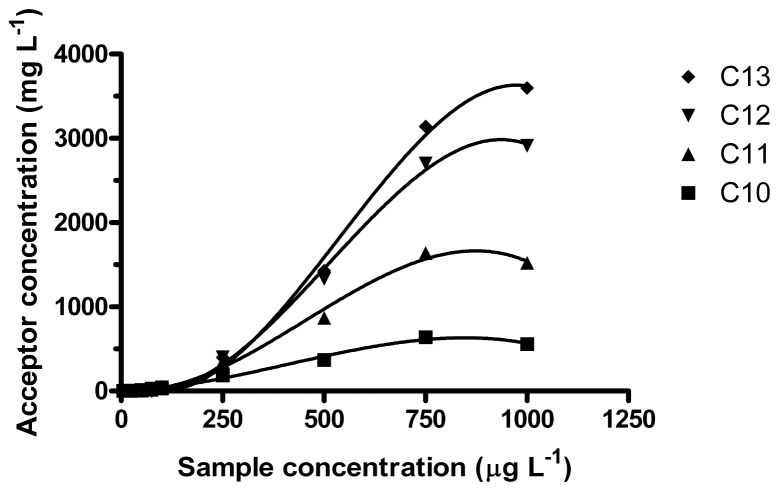
Acceptor concentration as a function of sample concentration over a wide LAS concentration range (*n* = 3). Conditions as Figure 1, except 100 mL sample extracted during 20 h at 550 rpm.

Regardless of sample volume, it was observed that enrichment was not constant over the studied sample concentration range. In the interval 5–1000 μg L^−1^, linear R^2^-coefficients for the acceptor concentration ranged from 0.934 (C_10_) to 0.9745 (C_13_) and the intercepts were significantly below zero. Better correlations were obtained by fitting the data to 3^rd^ order equations, giving R^2^-coefficients from 0.9925 (C_10_) to 0.9969 (C_12_) (Figure 3).

It was found that for lower sample concentrations (<150 μg L^−1^), the degree of enrichment was reversed compared to higher sample concentration (1 mg L^−1^), *i.e.*, for low concentrations C_10_ was enriched the most and C_13_ the least. As the sample concentration increased, the higher homologues were eventually enriched more. C_11_ surpassed C_10_ about 150–200 μg L^−1^, C_12_ surpassed C_11_ ∼250 μg L^−1^ and C_13_ surpassed C_12_ about 350–400 μg L^−1^ (100 mL samples, 20 h at 550 rpm). Furthermore, here it was also observed that the equilibration time was higher for 100 mL samples than for 250 mL samples. Unlike the equilibrium observed after 15 h for 250 mL samples, the enrichment was increasing for all homologues, except possibly C_10_, even at 24 h.

Non-linear extraction in ion-pair LPME of (cationic) surfactants (between 1 and 60 μg L^−1^) was also recently observed [34]. This was explained by adsorption of the cationic surfactants on the glass. However, anionic surfactants such as LAS are not expected to adsorb on glass or silica at pH 7, due to the negative charge of both silica surface and LAS [37]. This leads to the question whether the enrichment in ion pair LPME of surfactants is dependent on analyte concentration. In reversed micelle assisted protein extraction, it was reported that at low surfactant concentrations, mass transfer over the membrane interface limited extraction, while at higher surfactant concentrations, mass transfer through the membrane was limiting [37]. In extraction of polyamines, it was observed that *E*_e_ increased when the surface tension toward the acceptor decreased (by increasing the carrier concentration or decreasing the acceptor pH) [25]. Possibly, THA decreases the surface tension likewise here. In the current work, after the LAS:THA ion pair has been broken up in the acceptor, neutral THA may be back-extracted and diffuse back to the sample side of the membrane, where THA can once again be protonated. At any given time, THA back-extraction would depend on the already achieved *E*_e_. LAS ion pair formation and extraction with THA molecules that are already situated at the sample/membrane interface is probably faster than likewise pair formation and extraction from the bulk of the sample solution. Thus, it overall seems reasonable that enrichment in ion-pair LPME of LAS is dependent on sample concentration.

**Figure 4:**
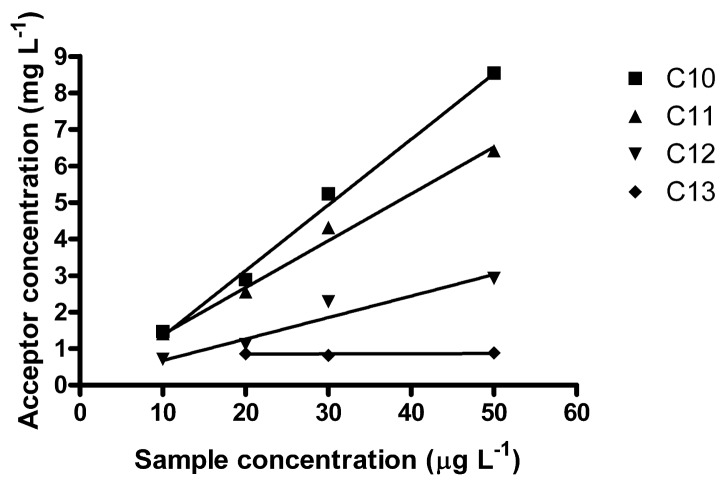
Acceptor concentration as a function of low LAS sample concentrations. Conditions as Figure 1 with *n* = 3 for 10 and 20 μg L^−1^ and *n* = 1 for 30 and 50 μg L^−1^. *E*_e_ was 152 for C_10_, 135 for C_11_ and 64 for C_12_. No *E*_e_ could be determined for C_13_ in this interval.

A non-linear curve may appear linear in a limited range, *i.e.*, the studied method might find practical use in a more limited concentration range. In the lower interval 10–50 μg L^−1^, which in fact is more environmentally relevant, better linear correlations were obtained (Figure 4). It may be noted that the R^2^-coefficients decreased with increasing homologue number; 0.9940 for C_10_, 0.9933 for C_11_, 0.9561 for C_12_ and 0.0811 for C_13_. *E*_e_ was 152 for C_10_, 135 for C_11_ and 64 for C_12_. For C_13,_ no reliable *E*_e_ could be determined. C_13_ also elutes last in the HPLC, exhibiting the largest band broadening. Average *E* in this interval was 0.63% (C_10_), 0.56% (C_11_) and 0.27% (C_12_) and 0.20% (C_13_) (which is rather low).

### 3.5. Matrix Effects

**Figure 5:**
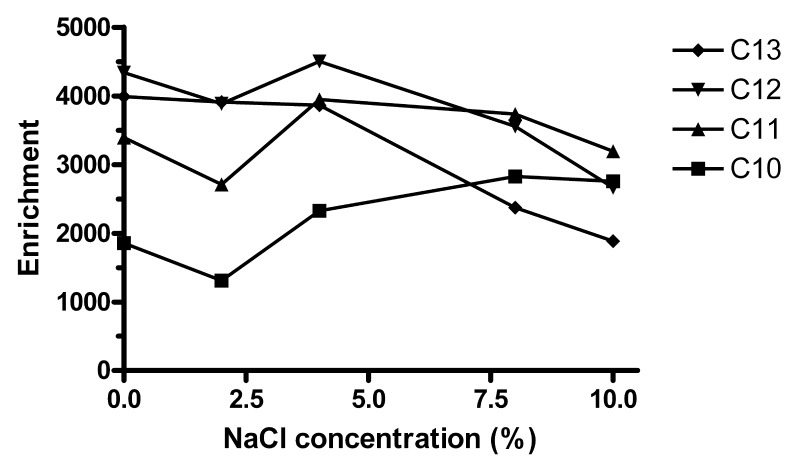
Effect of sodium chloride. Conditions as in Figure 1 (*n* = 1).

When the ionic strength of water is increased, the partitioning of hydrophobic solutes into lipophilic phases increases [2], but attraction between any pair of specific ions decreases. On adding up to 10% (*w/v*) NaCl to the sample, *E*_e_ of C_10_ increased slightly while the extraction of C_12_ and C_13_ decreased (Figure 5). C_11_ was approximately constant. Further extractions were made without addition of NaCl.

In tap water spiked to 1 mg L^−1^, all homologues were negatively affected (Table 2). However, C_10_ was affected the most and C_13_ the least. This was the opposite trend to the effect of NaCl. Anionic surfactants precipitate partly with divalent cations [2] and the local tap water contained 21–22 mg L^−1^ calcium and 1–2 mg L^−1^ magnesium (as measured by flame atomic absorption), which could influence the extraction. However, LAS sensitivity to water hardness usually increases with homologue number [2].

**Table 2:** Relative *E*_e_ due to matrix (*n* = 3) and method performance under standard addition in surface water (*n* = 5). Conditions as in Figure 1, except Figure 6 conditions and nominal LAS sample concentration 100 μg L^−1^ for humic acids. *E*_e_ in surface water was calculated as an average of the slopes in the standard addition curves. LOD refer to individual homologues. Precision (RSD) was determined by extraction of effluent samples nominally spiked to 10 μg L^−1^.

**Homologue**	**Relative *E*_e_ (%) due to matrix**		**Performance in surface water**		

	**Tap water**	**2.5 mg L^−1^ humic acid**	**Absolute *E*_e_**	**LOD (μg L^−1^)**	**RSD**
C_10_	8	57	11	0.7	12
C_11_	14	61	14	1.2	13
C_12_	33	61	12	1.2	17
C_13_	75	34	nd	4.6	nd

nd = not determined.

**Figure 6:**
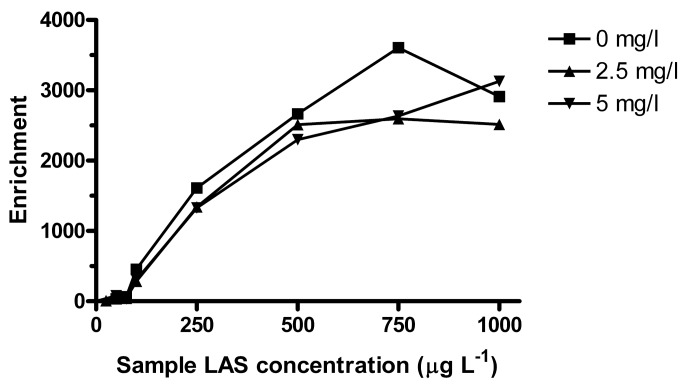
Effect of humic acids, exemplified with C_12_, over a wide LAS concentration range (*n* = 3). Conditions as Figure 1, except 100 mL sample extracted during 20 h at 550 rpm.

LAS can bind to dissolved organic matter (DOM), which decreases the freely dissolved concentration and affects the bioavailable amount [38,39]. It was observed that lower concentrations could be extracted when surface water samples were filtered prior to spiking (data not shown). The turbidity of the environmental samples was visually similar to that of 2.5 mg L^−1^ solution of Aldrich humic acids. Turbidity also depends on inorganic colloids, but in a simplified approach, the effect of organic matter on extraction was tested at 2.5 and 5 mg L^−1^ of humic acids. Based on reported log *K*_DOC_ for LAS to Aldrich humic acids_,_ the bound fraction (for isomers substituted at the second alkyl carbon) at 2.5 mg L^−1^ of humic acids would be about 4% for C_10_, 7% for C_11_, 17% for C_12_ and 44% for C_13_ [39]. Here, a larger effect was observed at LAS concentrations <100 μg L^−1^, where the average relative *E*_e_ compared to humic acid free sample was ∼60% for C_10_–C_12_ and 34% for C_13_ (Table 2). Between 100 and 500 μg L^−1^ LAS there was a slight decrease in enrichment due to humic acids, but no significant effect was seen above 750 μg L^−1^ (Figure 6).

### 3.6. Application to Environmental Samples

**Figure 7:**
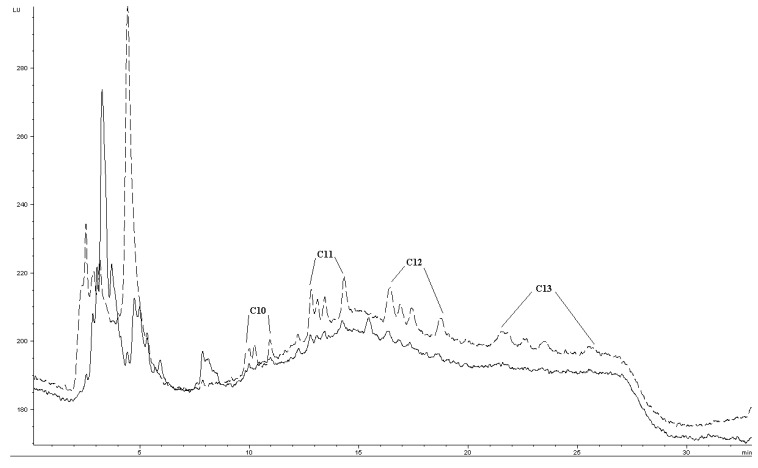
The lower chromatogram is obtained from an extract from sewage treatment plant (STP) effluent spiked with 25 μg L^−1^ and the upper chromatogram (dashed line) represents a 1 mg L^−1^ standard. Given concentrations refer to nominal concentrations and not to the concentration of each isomer peak. Conditions as Figure 1.

Quantification of LAS in surface water was attempted by standard addition from 5–50 μg L^−1^. In spiked environmental samples, *E*_e_ was about 7–19% of *E*_e_ in buffered reagent water (comparing Figure 4 and Table 2). The decrease may be due to matrix effects such as DOM (Table 2) or divalent cations, as the river water contains about 75–85 mg L^−1^ calcium and 7–8 mg L^−1^ magnesium (previously determined by titration). Precision was evaluated by extraction of five STP effluent samples spiked to 10 μg L^−1^. For C_10_, C_11_ and C_12_, precision was quite similar to buffered reagent water (Table 1 and Table 2). Method LOD for C_10_, C_11_ and C_12_ were estimated to about 5 μg L^−1^, for each individual group of homologues and based on nominal LAS concentration. Taking into account the estimated fractions of homologues, this gives individual homologue LODs ∼1 μg L^−1^. C_13_ had higher method LOD, due to its generally inefficient extraction at concentrations <50 μg L^−1^ and band broadening. C_13_ could be barely detected as very small peaks in an effluent sample nominally spiked to 25 μg L^−1^. This extract chromatogram is presented in Figure 7, together with a chromatogram of a standard to illustrate the general separation.

For the effluent, C_10_ and C_11_ were detected in all non-spiked aliquots and C_12_ in five of six such replicates. For the river samples, only one of the non-spiked samples (after the STP) contained LAS. However, all these concentrations were too close to LOD for certain quantification.

## 4. Conclusions

Ion-pair mediated LPME was studied for the anionic surfactant LAS in aqueous samples. Three matters were especially observed. Firstly, as an LPME method, the extraction was slow. Secondly, the degree of enrichment depended on sample concentration. Thirdly, lower homologues were enriched more efficiently at low sample concentrations with reversed conditions at higher concentrations. The mass transfer over the sample/membrane interface seems to be limiting. Possibly, analyte surfactants themselves influence the mass transfer at the SLM surface by decreasing the surface tension, or counter ion molecules that have already participated in extraction of LAS molecules enhance the extraction rate as the amine diffuse to the sample/membrane surface again. The observations on LAS extraction may be useful in LPME of other surfactants, which may be associated with higher risks than LAS itself.

For future investigations, it would be advantageous to measure the surface tension between the membrane and the sample solution or even between the membrane and the acceptor. It would aid if enrichment can be increased further at environmentally relevant concentrations, to remove the noise associated with baseline concentrations. For C_13_, this is crucial. The sample volume can probably be increased without the loss in precision we observed. Effects of organic matter may be reduced by addition of methanol or by filtration. If it is sufficient to report homologue concentrations and not isomers, there is also the possibility of using chromatographic methods that keep the isomers together in individual homologue peaks, thus giving higher homologue signals. Using MS detection and better columns, e.g., sub 2 μm particles, would also improve sensitivity.
